# Transcriptome Analysis of Floral Buds Deciphered an Irregular Course of Meiosis in Polyploid *Brassica rapa*

**DOI:** 10.3389/fpls.2017.00768

**Published:** 2017-05-12

**Authors:** Janeen Braynen, Yan Yang, Fang Wei, Gangqiang Cao, Gongyao Shi, Baoming Tian, Xiaowei Zhang, Hao Jia, Xiaochun Wei, Zhenzhen Wei

**Affiliations:** ^1^School of Life Sciences, Zhengzhou UniversityZhengzhou, China; ^2^Institute of Horticultural Research, Henan Academy of Agricultural SciencesZhengzhou, China

**Keywords:** polyploidy, meiosis, RNA-seq analysis, transcriptome, floral buds, *Brassica rapa*

## Abstract

Polyploidy is a fundamental process in plant evolution. Understanding the polyploidy-associated effects on plant reproduction is essential for polyploid breeding program. In the present study, our cytological analysis firstly demonstrated that an overall course of meiosis was apparently distorted in the synthetic polyploid *Brassica rapa* in comparison with its diploid progenitor. To elucidate genetic basis of this irregular meiosis at a molecular level, the comparative RNA-seq analysis was further used to investigate differential genetic regulation of developing floral buds identified at meiosis between autotetraploid and diploid *B. rapa*. In total, compared to its diploid counterparts, among all 40,927 expressed genes revealed, 4,601 differentially expressed genes (DEGs) were identified in the floral buds of autotetraploid *B. rapa*, among which 288 DEGs annotated were involved in meiosis. Notably, *DMC1* identified as one previously known meiosis-specific gene involved in inter-homologous chromosome dependent repair of DNA double stranded breaks (DSBs), was significantly down-regulated in autotetraploid *B. rapa*, which presumably contributed to abnormal progression during meiosis I. Although certain DEGs associated with RNA helicase, cell cycling, and somatic DNA repair were up-regulated after genome duplication, genes associated with meiotic DSB repair were significantly down-regulated. Furthermore, the expression of randomly selected DEGs by RNA-seq analysis was confirmed by quantitative real-time PCR analysis in both *B. rapa* and *Arabidopsis thaliana*. Our results firstly account for adverse effects of polyploidy on an entire course of meiosis at both cytological and transcriptomic levels, and allow for a comprehensive understanding of the uniformity and differences in the transcriptome of floral buds at meiosis between diploid and polyploid *B. rapa* as well.

## Introduction

Polyploidization has a distinctive role in evolution and speciation of plants and animals. Fundamentally the increase in ploidy level is less abundant in animals, but is more frequently observed in the majority of plant taxa (Sundstrom et al., 2008), with over 30–80% of angiosperms undergoing chromosomal duplication during their evolutionary history (Masterson, 1994; Comai, 2005; Paterson, 2005; Soltis et al., 2009; Jiao et al., 2011). To survive and compete with its diploid progenitor, the neopolyploids must overcome genetic instability occurring during meiosis and environmental pressures (Ramsey and Schemske, 2002; Comai, 2005). Polyploids may arise by intraspecies genome duplication (autopolyploids) or via interspecific hybridization (allopolyploids). The polyploidy-associated effects have been extensively reviewed in regard to expression patterns, environmental stress and chromosomal behavior, to explain changes between the established polyploids and diploid ancestors (Bomblies et al., 2015; Lloyd and Bomblies, 2016).

Meiosis is distinguished from canonical mitotic division by two events, in which correct pairing of homologous chromosomes are the first essential feature during meiosis, and the second event is involved in two sequential rounds of cell division, to reduce the chromosome set of diploid cells to haploid gametes. Therefore, a well-established course of meiosis are not only essential for polyploid reproduction itself, but also crucial for genetic stability in polyploid species (Schuermann et al., 2005). One adverse consequence of polyploidization is the imbalanced segregation of homologous chromosomes during meiosis (resulting in genomic instability) and partially leads to pollen sterility and less fertility for polyploid species. To overcome this phenomenon, the evolved polyploids have established genetic stability by losing their polyploidy traits and exhibiting diploidy traits through the process of diploidization, as in the case of palopolyploids (Mitchell-Olds and Clauss, 2002). For instance, the natural autotetraploid *Arabidopsis arenosa* has adapted to whole genome duplication by the reduction of chiasmata formation (Yant et al., 2013). Fewer chiasmata formation limits the crossover frequency to one per chromosome which prevents multivalent association. Despite numerous consequences, the evolved polyploid species also display various advantages in phenotypic and genetic traits such as genetic diversity, increase in cell volume, increase in agronomic traits, and resistance to diseases (Marhold and Lihová, 2006; Li et al., 2012). Pecinka et al. (2011) has examined the rate of meiotic recombination in male and female gametes in polyploid *Arabidopsis* and concluded that the rate of meiotic recombination increased in tetraploids, and thus inferred that genome doubling probably results in rapid creation of genetic diversity in polyploid species (Madlung and Wendel, 2013).

In the case of newly formed allopolyploids, the pairings of homoeologous chromosomes were frequently observed during meiosis, giving rise to interchromosomal rearrangements and epigenetic modifications (Chester et al., 2012; Bottley, 2014). Recently, the transcriptome analyses utilizing flower tissues or anthers, have identified numerous genes involved in meiosis (Deveshwar et al., 2011; Libeau et al., 2011). Furthermore, the transcriptome analysis of the isolated male meiocytes was performed to reveal insights into the regulating pathways related to meiosis in several plants including *Arabidopsis* (Chen et al., 2010; Yang et al., 2011), Maize (Dukowic-Schulze et al., 2014), sunflower (Flórez-Zapata et al., 2014), which generally provided an overview of the gene expression profiling specific to development of male meiocytes in plants.

In the present study, we sought to better understand the polyploidy-associated effects on development of plant reproductive tissues (immature floral buds) during meiosis at both cytological and molecular levels in the synthetic autotetraploid *Brassica rapa* after their establishment in comparison with its diploid progenitors. The immature floral buds were firstly subject to cytological analysis, which demonstrated that the chromosomal behavior was severely distorted during meiosis in the autotetraploid *B. rapa*, and to elaborate on molecular basis of this cytological adversity, the immature floral buds identified at meiosis were subjected to RNA-seq analysis to investigate differentially expressed genes (DEGs) involved in meiosis between autotetraploid and diploid *B. rapa* at the transcriptome level. In general, our results provide full insights into polyploidy-associated effects on meiosis at both cytological and transcriptomic levels, and especially allow for a profound understanding of the uniformity and differences in the transcriptome of reproductive immature floral buds between diploid and polyploid *B. rapa*.

## Materials and methods

### Sample collection

Both autotetraploid and diploid *B. rapa* were grown under green-house conditions of 16 h light and 8 h dark photoperiod, at temperatures of 22°C daytime and 18°C nights. The immature floral buds were firstly harvested and then cytologically identified at meiosis, and immediately frozen and kept in liquid nitrogen in three biological replicates until use.

### Cytological analysis

For the investigation of chromosomal behavior, the immature flower buds approximately 1.0–1.5 mm (identified at meiosis) were fixed in Carnoy's Fluid (alcohol: glacial acetic acid, 3:1) for 4 h and stored in 70% ethanol at 4°C until use. Fixed buds were rinsed with distilled water (3 × 3 min), and then washed with citrate buffer (10 mM, pH4.5) (2 × 5 min). The sample was then incubated for 1 h at 37°C in an enzyme mix containing pectolase (0.5% w/v) and cellulose (0.5% w/v), in citrate buffer. Chromosome spreads were prepared as previously described elsewhere (Leflon et al., 2006; Nicolas et al., 2009) with minor modifications. Upon chromosomal analysis, PI solution of 10 μg/mg was applied to the prepared slides. Chromosome behavior was observed during meiosis for both diploid and autotetraploid *B. rapa*, assisted with the Olympus BX53 epifluorescence microscope equipped with cooled CCD DP73 digital camera (Japan, Olympus-lifescience).

### RNA extraction and RNA sequencing

TriZol (Invitrogen) was used to isolate total RNA of the immature floral buds according to the manufacturer's instructions with minor modifications. Quantity and quality of the extracted RNAs were assessed by the Nanodrop ND1000 spectrophotometer (Nanodrop Technologies), and the A260/280 and A260/230 ratios were calculated to determine the purity of the isolated RNA. The experiment was conducted with three biological replicates for the immature floral buds collected from both diploid (T1, T2, T3) and autotetraploid (T4, T5, T6) *B. rapa*. Preparation of library construction was performed by the Institute of Biomarker (Beijing, China). Such procedures were conducted fragmentation of RNA, cDNA synthesis, PCR amplification, and construction of libraries for RNA-seq. The cDNA libraries were sequenced using the Illumina HiSeq 2000 platform (Illumina, California USA). Finally, the raw data images were transformed into sequencing information by base calling and stored as FastQ format files. The RNA-seq data in fastQ format have been deposited in the NCBI Sequences Read Archives (SRA) with accession number SRP104015.

### Data analysis: quality control and mapping of raw reads

Raw data were filtered and trimmed by removal of reads with adapters, reads that contain unknown bases of more than 10% and reads with low quality base score of <5. The BRAD database (http://brassicadb.org) was employed to download the references genome for alignment purposes, and the sequence file used was *B. rapa* genome sequence version v1.5. The clean and filtered reads were aligned to the references genome using TopHat2 (v2.1.1; Kim et al., 2013). The Cufflinks suite (v2.1.0) was utilized on mapped reads to construct transcripts and to measure expression of transcripts by the quantification of FPKM-values (Trapnell et al., 2012).

### Identification of differentially expressed genes (DEGs)

Count data values were normalized before dataset was run by the R program. DEGs were determined by using the DESeq program with a log-fold expression change (log FC) of ≥2 and False Discovery Rates threshold of <0.01. The FDR of DEGs was based on *p* < 0.05 which was adjusted by the Benjamini-Hochberg correction method (Anders and Huber, 2010).

### Functional annotation of DEGs

BLAST similarity searches for gene models were search against NCBI non-redundant protein (Nr) database. UniProtKB\Swissprot database was also searched. The transcripts that aligned to the KOG (Eukaryotic Orthologous Groups) database were classified according to functions of transcripts (http://www.ncbi.nlm.nih.gov/COG/). Blast2GO v2.5.0 (https://www.blast2go.com/) was performed to assign Gene Ontology (GO) terms. Further, annotation of the genes for GO mapping was restricted to significant BLASTX hits below e value 1e-03, with a score of 50 as the annotation cut-off and 5 for the GO weight. Kyoto Encyclopedia Genes and Genomes (KEGG) mapping was used to annotate the pathway of the transcripts.

### Expression verification of DEGs in both *B. rapa* and *A. thaliana*

Expression levels of 24 DEGs were determined by quantitative real-time PCR (qRT-PCR) in diploid and autotetraploid *B. rapa*. TriZol reagent was used to extract total RNA of immature floral buds according to the manufacturer's instructions with 1 μg of the purified sample reverse transcribed. SuperScript III Reverse Transcriptase (Invitrogen) kit was utilized to construct the first strand of cDNA using oligo (dT) primers. For qRT-PCR analysis 1 × SYBR master kit was used with 0.8 μl of cDNA template and 0.4 μl of PCR primers. Gene specific primer sequences were designed by Primer premier 5.0 (PREMIER Biosoft) with amplification fragments between 100 and 200 bp, and the specificity of primer pairs was checked with Blast primer design tool (NCBI; Table S1).

Orthologous genes in *Arabidopsis thaliana* were generated using the Gramene webpage (http://gramene.org/), to organize a list of homologs between *B. rapa* and *A. thaliana* (Table S2). The isolation, synthesizing of cDNA, and primer design methods were similar to the qRT-PCR validation procedure as mentioned above. Relative expression of interested DEGs was normalized by β*-actin* and analyzed using the 2^−ΔΔCT^ method. The qRT-PCR was carried out utilizing the QIAGEN ROTOR gene 6000 (QIAGEN), and each reaction was performed in triplicates.

## Results

### Aberrant course of meiosis in autotetraploid *B. rapa*

**Figure 1:**
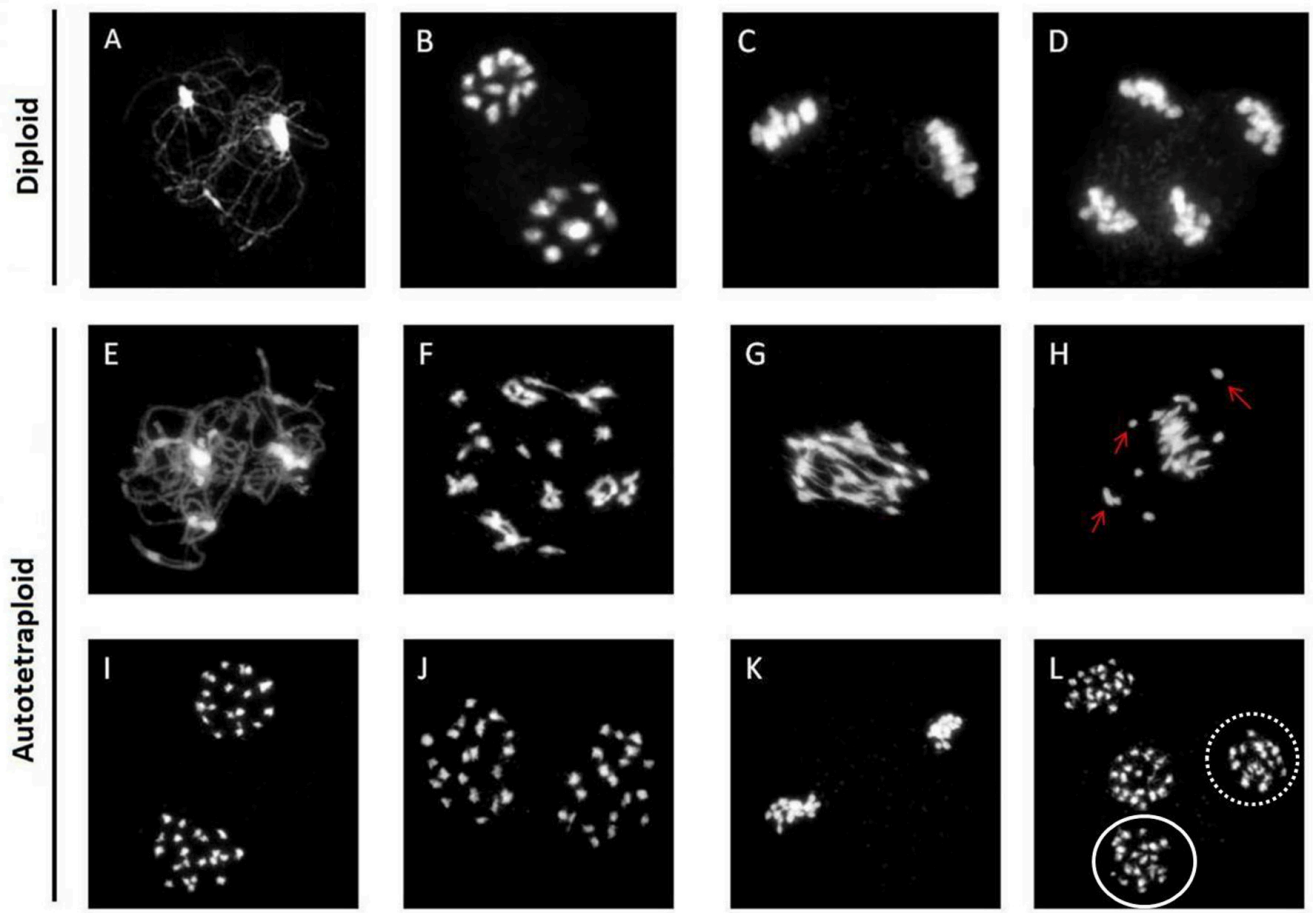
**Abnormal chromosome behavior in the synthetic autotetraploid *B. rapa*.** Normal chromosomal behavior displayed in the diploid *B. rapa* from early prophase to anaphase II **(A–D)**, and normal chromosome morphology through early prophase and metaphase I was also observed in autotetraploids **(E–G)**. However, a subset of cells consisting of multivalents and univalents (arrows) chaotically dispersed at metaphase I **(H)**. Homologous chromosomes segregate equally during anaphase I forming two polar groups of chromosomes **(I)**. Unequal segregation of chromosomes in the ratio of 19: 21 at anaphase I **(J)**, Normal alignment of chromosomes in cell at metaphase II **(K)**, but cells at anaphases II consist of unequally segregated chromosomes **(L)**. Dotted oval consist of 19 chromosomes while solid oval has 21 chromosomes. Bar = 10 μm.

**Figure 2:**
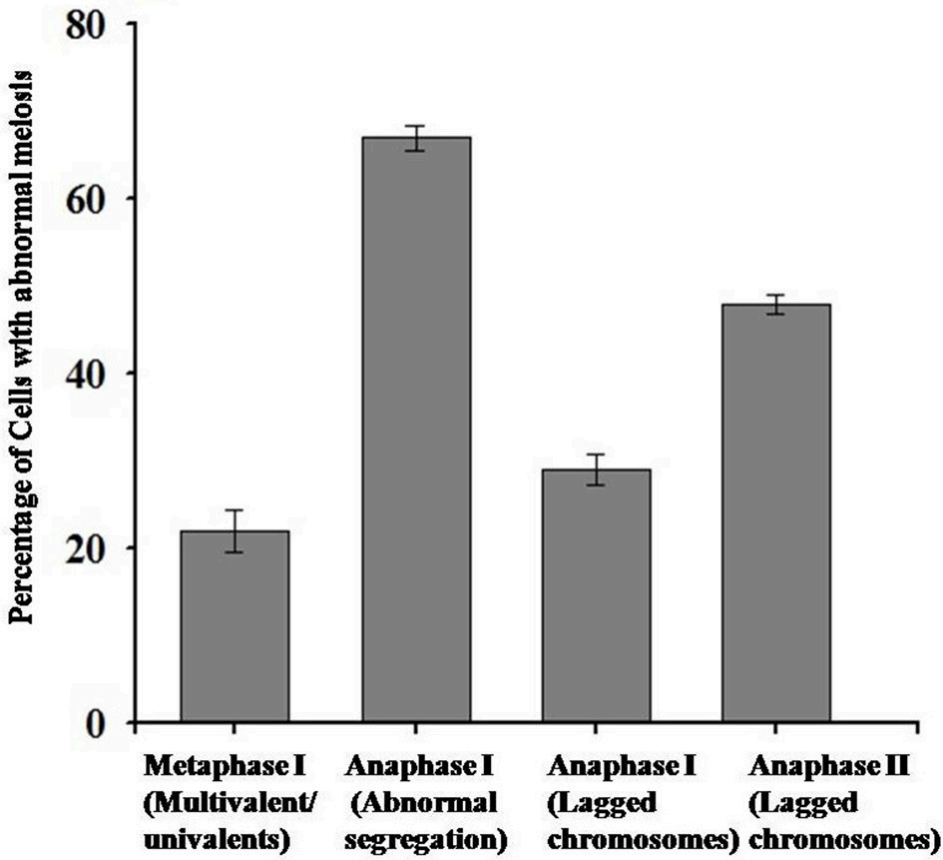
**Statistical analysis of abnormal behavior of chromosomes during meiosis in autotetraploid *B*.* rapa***.

Chromosomal behavior at meiosis was firstly analyzed in both diploid and synthetic autotetraploids *B. rapa*. As shown in Figure 1, homologous chromosomes were completely synapsed at pachytene, paired as bivalents at metaphase, and equally separated at anaphase in diploid *B. rapa* (Figures 1A–D). However, the chromosome pairing in autotetraploid *B. rapa* was typically influenced by the presence of more than two sets of homologous chromosomes. Compared with diploids, the autotetraploids differ significantly with the formation of multivalents and univalents at metaphase I, unequal segregation at anaphase I and anaphase II (Figures 1E–L). In autotetraploid *B. rapa*, the statistical analysis indicated that during anaphase I approximately 67% of observed PMC were segregated abnormally at ratios of 19:21 (46 cells), 18:22 (13 cells), and 17:23 (5 cells), and 48% of PMCs at anaphase I consist of the lagged chromosomes. Furthermore 29% of observed cells produced the lagged chromosomes at anaphase II and 22% of abnormal cells consist of multivalents and univalents at metaphase I in the synthetic autotetraploid *B. rapa* (Figure 2). These results indicated that after polyploidization, the whole course of meiosis was severely distorted in autotetraploid *B. rapa* in contrast with its diploid counterparts.

### RNA-seq, global assembling, and expression profiling of read data

**Figure 3:**
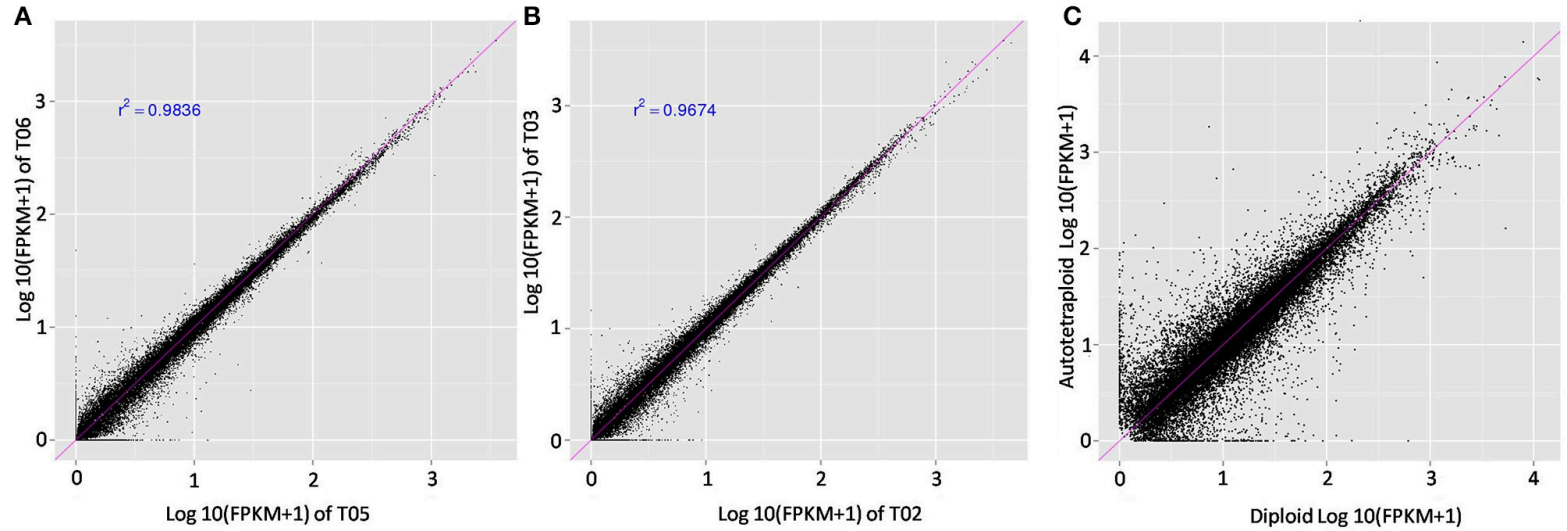
**Comparison of the FPKM values among the biological replicates**. Pearson correlation and scatter plot matrix of log_2_ normalized expression of the *Brassica rapa* expressed genes in autotetraploids (T04, T05, T06) and diploids (T01, T02, T03). The pink solid line is the identity line. Panels **(A,B)** were about correlation between biological replicates, the **(C)** showed correlation between the autotetraploid and diploid FPKM-values.

RNA-seq analysis was then used to investigate genetic alternations in autotetraploid *B. rapa* in contrast with diploids. Using Illumina sequencing, 29.07 GB of clean read data, a Q30-value no <88.55% and GC content between 46.88 and 47.50% were generated for all six libraries (Table S3, Figures S1, S2). For subsequent analyses, the clean reads were aligned to the reference genome with the autotetraploids generating an average of 74.34% of coverage and the diploids 75.88% coverage (Tophat2). Reads between 24.12 and 25.66% did not map to the reference genome. This specified that transcribed regions in the reference genome were unidentified, or putative transcripts mapped to genomic regions were not found inside of the annotated regions. To determine the expression level of genes, the mean expression patterns of sample sets based on threshold criteria of FPKM were analyzed, utilizing Cufflinks v2.2.1. In total 40,927 genes were identified with 1,001 genes being novel. The FPKM-values were compared to determine if the values significantly correlated (Figure 3). Comparison of the FPKM-values within sample sets indicates correlation with a few outliers, the correlation coefficient of *r*^2^ = 0.98 was constant between diploid and autotetraploid *B. rapa* for all biological replicates.

### Analysis of differentially expressed genes (DEGs)

**Figure 4:**
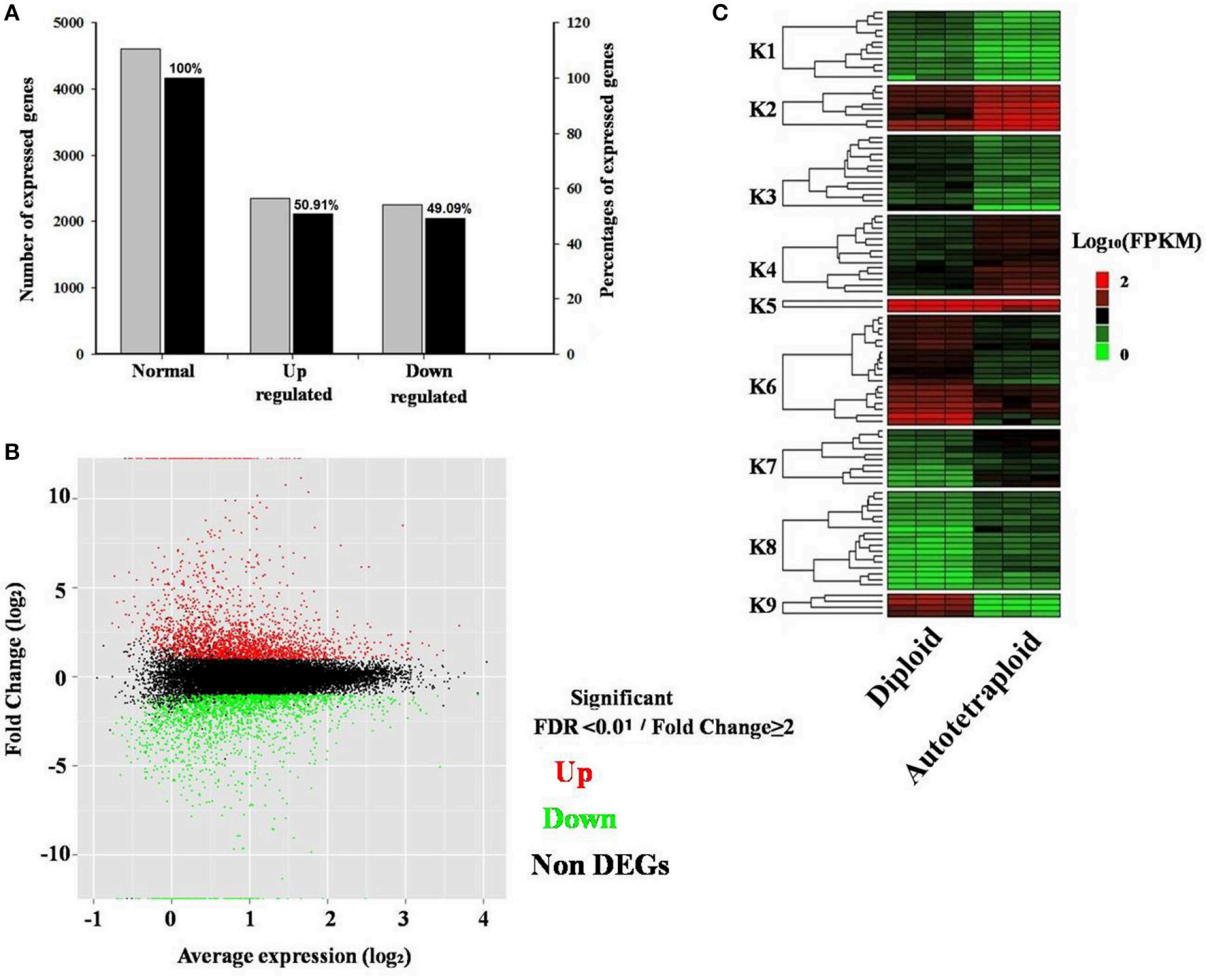
**Distribution and expression analysis of differentially expressed genes. (A)** Percentage of DEGs up-and down-regulated. **(B)** MA plot of RNA-seq data obtained from diploid and autotetraploid. Y-axis representing log_2_ fold change of all expressed genes vs. average expression normalized to log_2_ scale for each gene. **(C)** Heatmap analysis of a subset of DEGs across three biological replicates in autotetraploid and diploid *B. rapa*. Expressed genes are FPKM normalized log_10_ transformed values. Nine cluster are shown (K1–K9) from the subset of expressed genes.

The DEGs were detected based on the false discovery rate (FDR) of <0.01 and log-fold expression change (log FC) of ≥2. Among all 40,927 expressed genes revealed, a total of 4,601 genes were differentially expressed between autotetraploid and diploid *B. rapa* (Figure 4A), with 2,343 and 2,259 genes up-and down-regulated respectively (Figure 4B), which indicated a relatively small amount of DEGs (11.24%) were identified between diploid and autotetraploid *B. rapa*. Hierarchical clustering of the randomly selected DEGs was also performed to distinguish expression patterns between autotetraploid and diploid *B. rapa* (Figure 4C), and the results showed that the DEGs in cluster K1, K3, K6, and K9 were all down-regulated for the autotetraploids, but K2, K5, K7, K4, and K8 were significantly up-regulated for autotetraploids.

**Figure 5:**
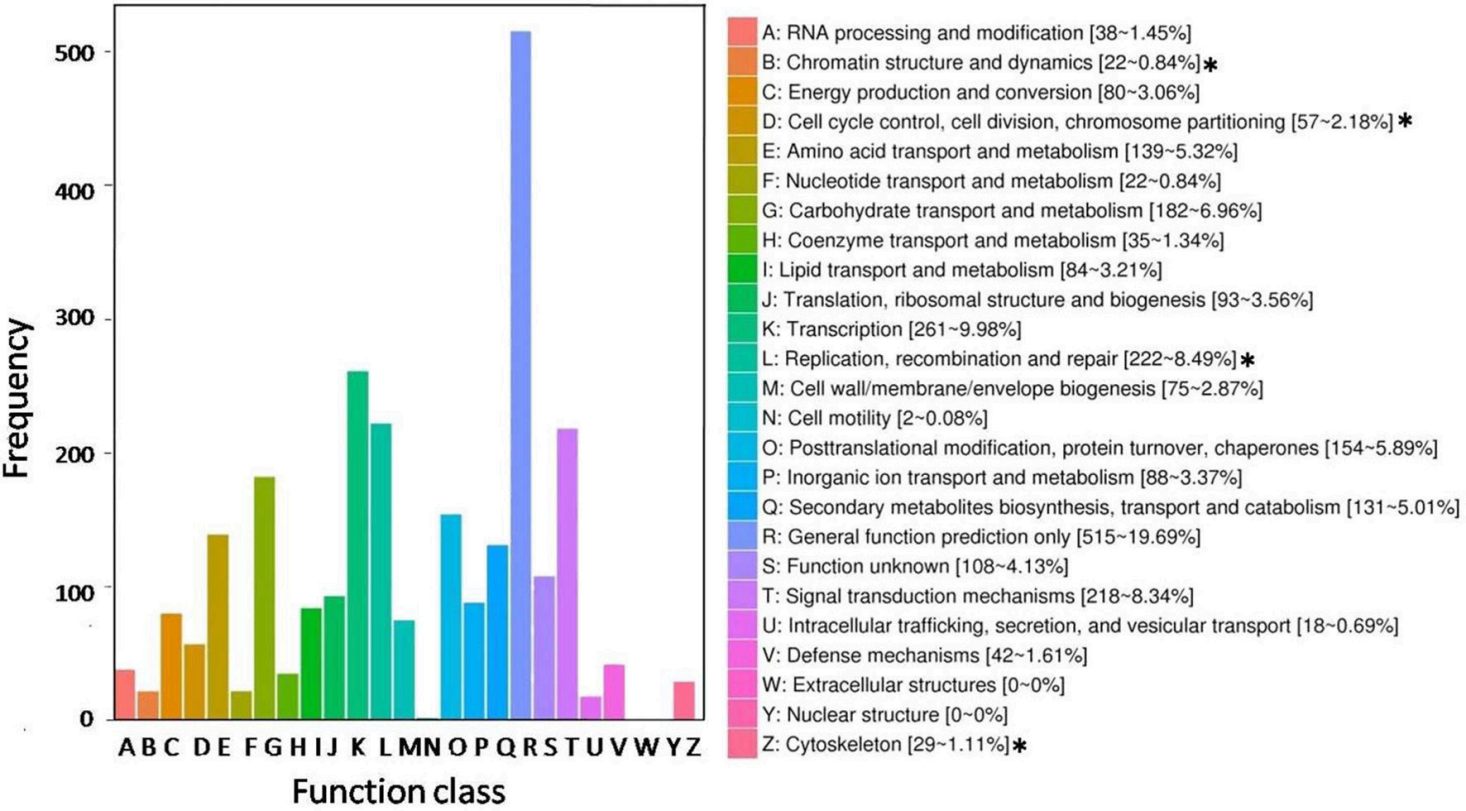
**Histogram representing clusters of orthologous genes (COG)**. Out of 4,602 transcript, 2,615 sequences were clustered in the various COG groups. The corresponding graph represents concentrated functional clusters with number and percentage of transcripts in parenthesis. The asterisks (^*^) represent functional clusters overrepresented for putative meiosis-related genes.

**Figure 6:**
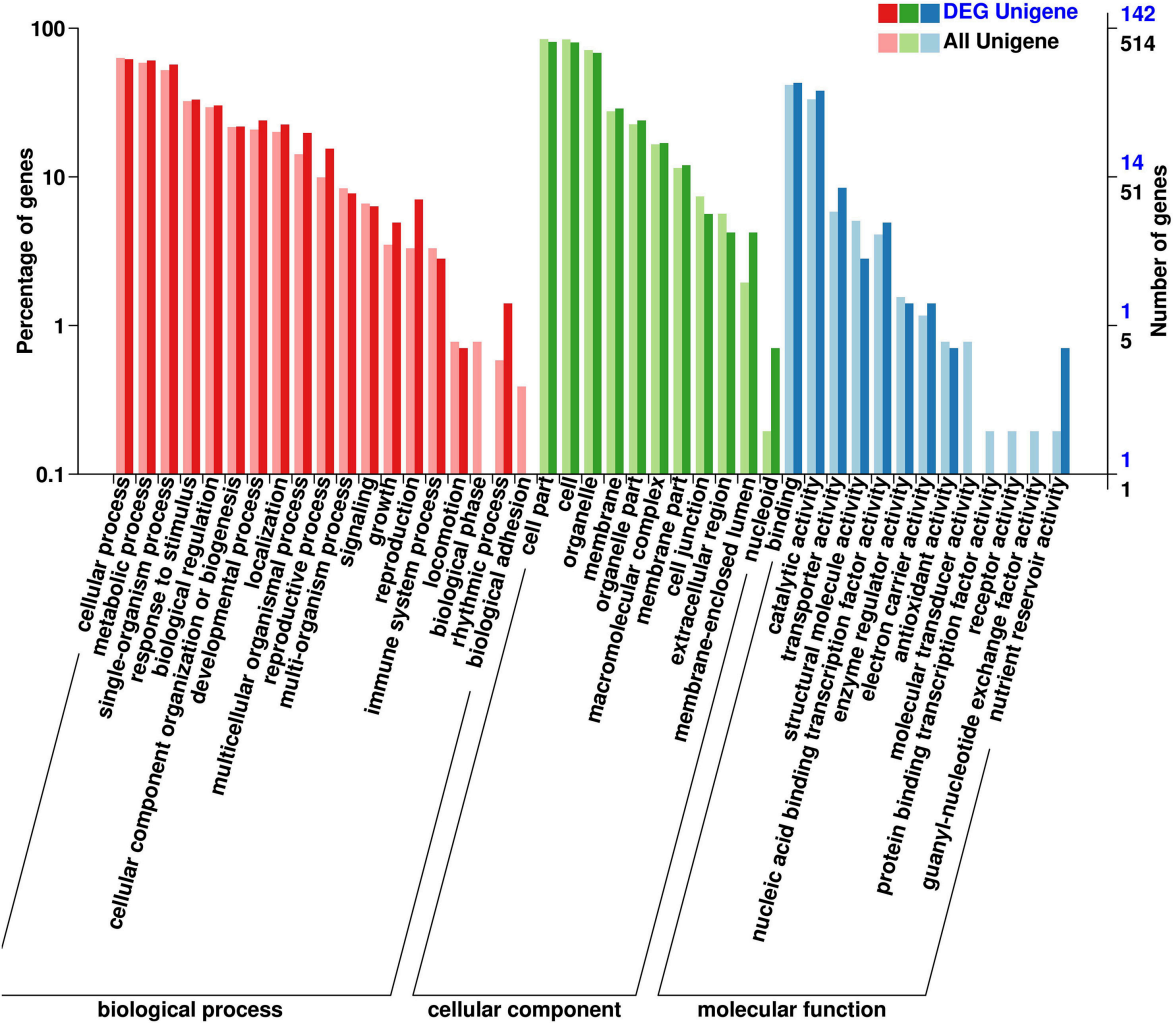
**Gene ontology classification of novel DEGs**. A total of 142 DEGs were classified into three groups of biological process, cellular component and molecular function, according to gene ontology.

**Figure 7:**
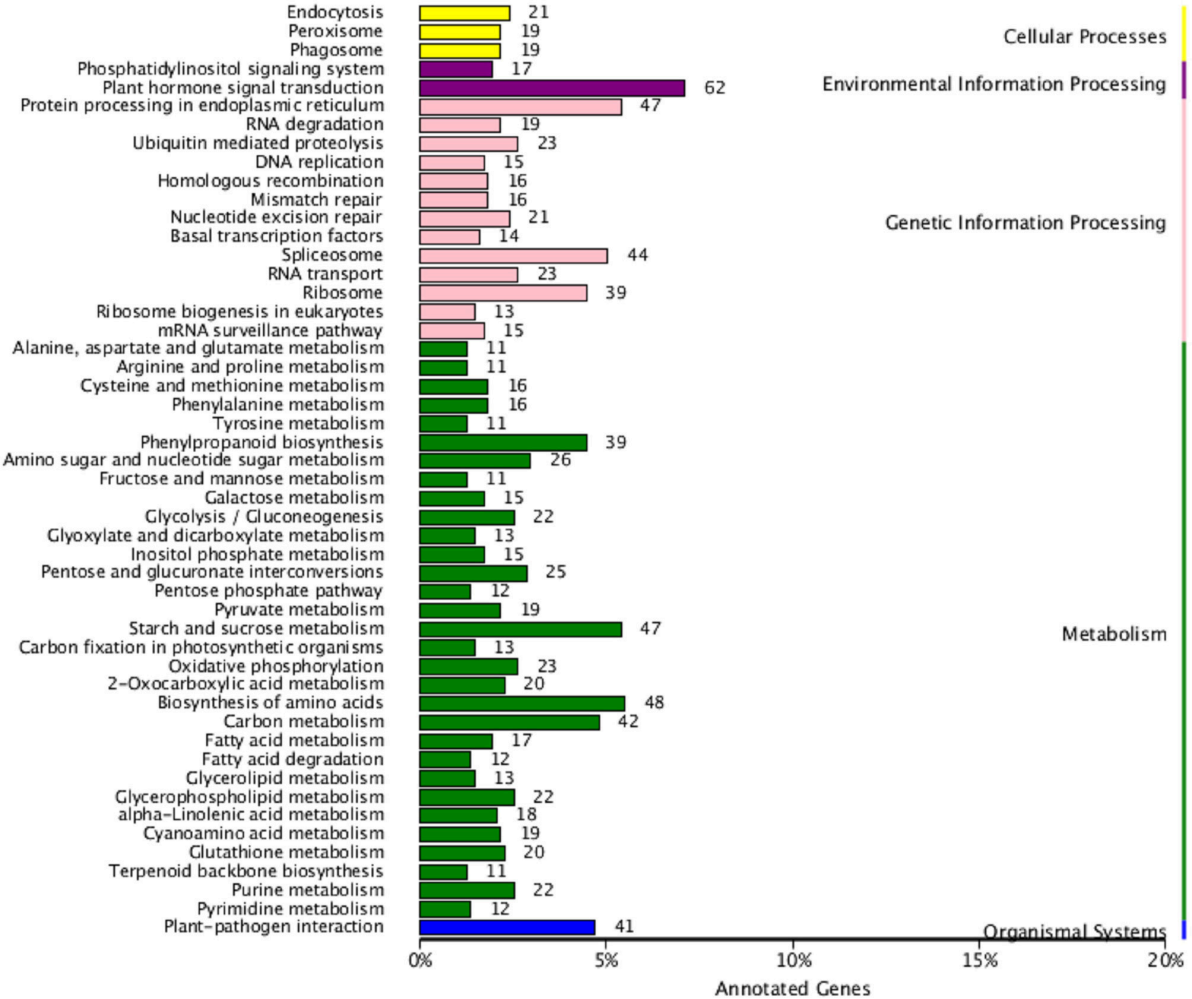
**DEGs enriched by KEGG pathways in autotetraploid *B. rapa***.

All DEGs were annotated using the COG classification, GO, KOG, NR, KEGG pathway and Swiss-Prot. About 97% of the DEGs were matched with at least one database (Table 1; Table S4). For COG classification, 2,615 of the 4,602 DEGs were functionally classified into 25 COG categories (Figure 5). The COG clusters revealed that replication, recombination, repair (222 DEGs, 8.49%), transcription (261 DEGs, 9.98%) and general function (515 DEGs, 19.69%) were overrepresented groups for DEGs, which correlated with results from the KEGG pathways. Gene Ontology analysis was conducted by mapping novel DEGs to the BLAST2GO database. According to GO classifications, most DEGs were clustered in cellular process (61.97%), reproductive process (15.49%), and binding (42.96%) (Figure 6). The biological functions of DEGs in autotetraploid *B. rapa* were further annotated to the KEGG biochemical pathways. Among the 4,601 DEGs identified, 1,453 genes were functionally assigned to 50 biological pathways with biosynthesis of amino acids (48 DEGs, 3.3%), plant hormone signal transduction (61 DEGs, 4.2%), and protein processing endoplasmic reticulum pathways (47 DEGs, 3.2%) substantially enriched (Figure 7).

**Table 1:** **Annotation of Differentially Expressed Unigenes**.

**Annotation tools**	**Number of genes annotated**
Cluster orthologus groups	1,718
Gene ontology	141
Kyoto encyclopedia of genes and genomes	1,453
KOG	2,257
NR	4,478
Swiss-Prot	3,382
Total genes annotated	4,481

### Analysis of DEGs concerned with meiosis

**Figure 8:**
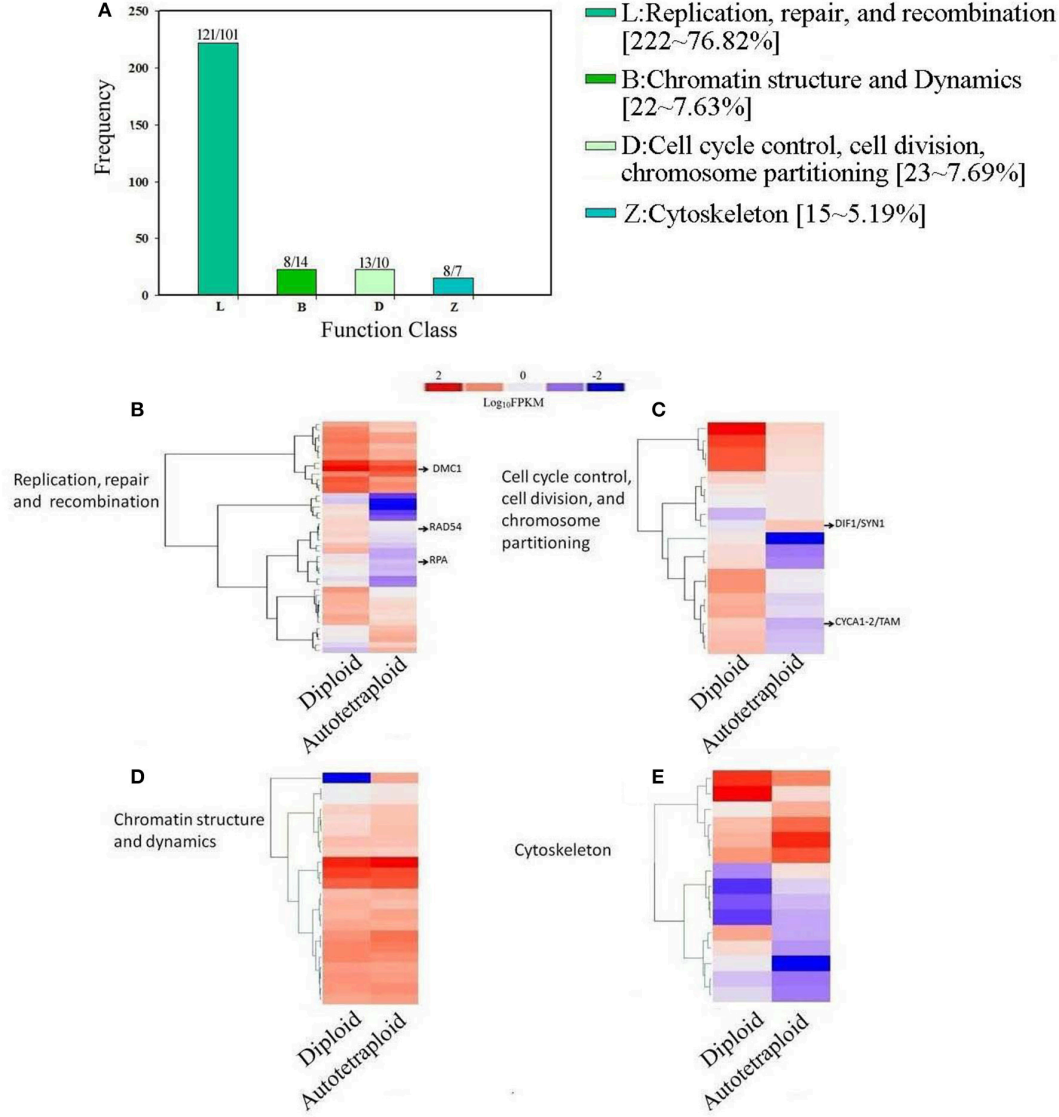
**Distribution and expression analysis of meiosis-related genes in COG classification. (A)** A number of up- and down-regulated meiosis-related genes clustered into 4 COG categories. **(B)** Overrepresented genes enriched in Replication, repair, and recombination, **(C)** Analysis of enriched genes in Chromatin structure and dynamics, **(D)** Overrepresented genes enriched Cell cycle control, cell division, chromosome partitioning **(E)** Z: Cytoskeleton. The log_10_-transformed FPKM-values range from 2 to −2.

To reveal genetic basis related to the altered meiotic course in autotetraploid *B. rapa* in comparison with diploids in Figure 5, the enriched COG categories were selected for sub-classification as they were apparently concerned with meiosis: (1) replication, recombination, and repair; (2) chromatin structure and dynamics; (3) cell cycle control, cell division, chromosome partitioning; and (4) cytoskeleton. Among the 4,601 DEGs, 288 genes were identified putatively related to meiosis (Figure 8A). Using the BLASTN search from the *Arabidopsis* database (TAIR), we further identified meiotic orthologous genes in *B. rapa*, and 11 known meiotic genes were identified and differentially expressed between diploid and autotetraploid *B. rapa* (Table S5).

As illustrated in Figure 8, the Cluster L representing for replication, recombination and repair was significantly enriched with 121 up-regulated genes and 102 down-regulated genes (Table S6), which were also subject to hierarchical clustering (Figure 8B), and the results showed that the known meiotic genes including *RAD54, DMC1*, and *RPA* were significantly down-regulated in autotetraploid *B. rapa*. In the Cluster D for cell cycle control, cell division, and chromosomes partitioning, the results showed that although 10 genes were down-regulated, 13 genes were up-regulated (Table S6), especially the known meiotic genes *DIF1/SYN1* and *Cyclin A1-2/TAM* were up-regulated (Figure 8C). In the Cluster B for chromatin structure and dynamics, although most genes had relatively similar expression pattern between autotetraploid and diploid *B. rapa* (Figure 8D), we found 7 DEGs were up-regulated which include *NF-YB8* gene known to regulate flower time and plant development and 14 DEGs down-regulated including histone related genes (H2AV and H4; Table S6). In the Cluster Z for cytoskeleton, we identified 8 genes were up-regulated, and 7 genes were down-regulated (Figure 8E), but we did not identify any known meiosis-related genes. Furthermore, we also identified 6 known meiosis genes not enriched in the above-mentioned COG clusters (Table S6). The known meiotic genes *MMD1* and *HOP2* were enriched in general function (R) (Figure 5), and *HOP2, XRI1, ZYP1a* and *ZYP1b* were not classified into any COG clusters.

In addition, the selected 288 DEGs were assigned to biological pathways using KEGG database, and among them 108 DEGs were functionally assigned to 28 pathways (Table S7), including meiosis-related pathways: homologous recombination (2.8%), DNA repair and recombination (6.5%), DNA replication (3.7%), chromosomes and associated proteins (4.6%) (Figure S3). Homologous recombination was essential for accurate repair of DNA double stranded breaks (DSBs) created by highly conserved SPO11 proteins during meiosis in most eukaryotes (Shingu et al., 2012), and known genes *DMC1, RAD54*, and *RPA*, as key mediators to regulate this process were significantly down-regulated in this pathway. The *Cyclin-A1-2/TAM*, known to regulate the progression of cell cycle during meiosis was the only cyclin-related gene within DNA replication pathway that was up-regulated (Jha et al., 2014). Besides, a portion of genes enriched were characterized as critical players in regulating transcription, DNA replication, DNA repair, and chromosome stability (Table S7). These results indicated as chromosome sets were doubled in autotetraploid *B. rapa*, most genes concerned with meiosis were likely up-regulated, or at least normally expressed to meet the demand of increased genomic contents during meiosis. Otherwise, the down-regulation of meiotic key genes would probably cause the disturbed chromosome behavior during meiosis in autotetraploid *B. rapa* in contrast with diploids.

### qRT-PCR analysis of meiosis-related genes

**Figure 9:**
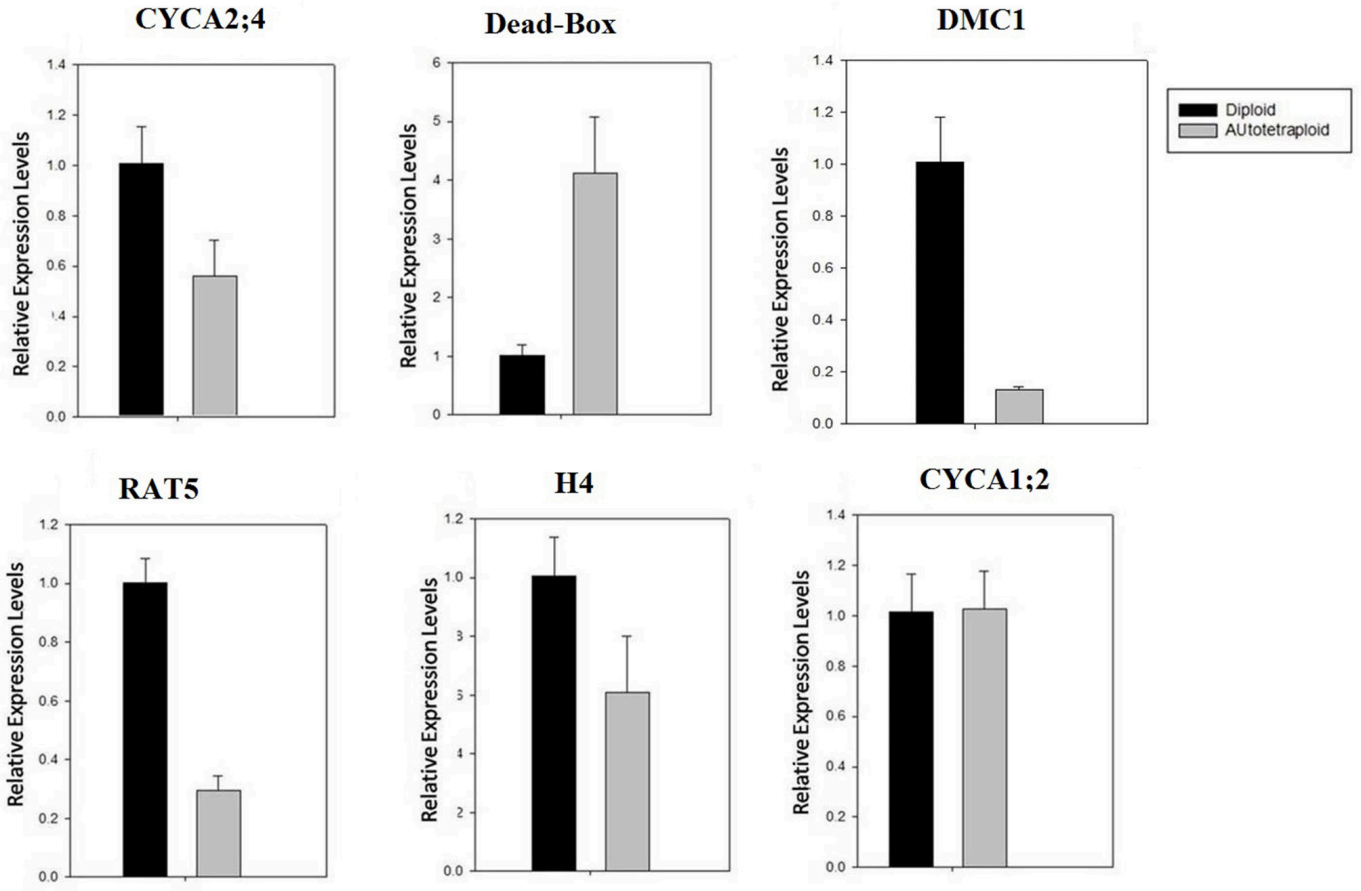
**qRT-PCR validation of expression of selected meiosis-related genes**. The relative expression was normalized by the comparison with β*-actin* expression. Notably, the expression of DMC1 was significantly down-regulated. Note: the expression level of other selected genes was also analyzed (see Figure S4).

Quantitative real-time PCR (qRT-PCR) analysis was performed for verification of expression pattern of 24 meiosis-related genes, to confirm consistence with the RNA-seq data in autotetraploid *B. rapa* in comparison with diploids. The results showed that more than 20 genes displayed a very similar expression pattern between qRT-PCR and RNA-seq data, though some genes did not positively correlate with each other (Figure 9, Figure S4). Furthermore, we also analyzed the expression level of their corresponding meiotic orthologous genes in *Arabidopsis*, to determine the reproducibility and reliability of these results in expression patterns in *B. rapa*. The qRT-PCR analysis showed that the overall expression patterns of meiosis-related genes were quite similar between *A. thaliana* and *B. rapa* (Figure S5).

## Discussion

### Known meiotic DSBs repair-related genes in autotetraploid *B. rapa*

Homologous recombination (cross-over formation) is essential for recognition, synapsis, and pairing of homologous chromosomes during meiosis in plants, and thus the involvement of meiotic genes is crucial for maintain stability of meiosis in polyploid plants (Armstrong et al., 2002; Cai et al., 2003; Higgins et al., 2005). Cross-over formation is initiated by the programmed DSBs at prophase I, which required the functioning of the conserved protein SPO11 (Keeney et al., 1997; Shingu et al., 2012). In the present study, we specifically found that SPO11 and DSBs formation associated genes displayed a similar expression level in both autotetraploid and diploid *B. rapa* (Table S8), which indicated that polyploidization may not affect formation of DSBs during meiosis in autetraploid *B. rapa*.

After initiation of meiotic DSBs induced by SPO11, the formation of cross-over between homologous chromosomes was promoted by meiosis-specific recombinase DMC1, which was dimerized with RAD51 and mediated via ASY1 for the DMC1-dependent pathway for homologous recombination during meiosis (Sanchez-Moran et al., 2008; Da Ines et al., 2013). In addition, the interaction of HOP2/MND1 complex would be activated during the DMC1 pathway (Petukhova et al., 2005; Vignard et al., 2007), to enhance single-stranded DNA invasion (Chi et al., 2007), and thus the accumulation of DMC1 was effected by the expression of either protein (Vignard et al., 2007; Uanschou et al., 2013). Moreover, RAD54 as an accessory factor, could interact with RAD51 and DMC1 (Raschle et al., 2004), and RPA as a heterotrimeric single stranded DNA-binding protein implicated in meiotic recombination (Soustelle et al., 2002; Osman et al., 2011), modulating the assembly of RAD51 and DMC1 filaments in ssDNA during meiotic DSBs repairing (Osman et al., 2011). In the present study, the comparative transcriptome analysis showed that in the DMC1-dependent pathway for DSBs repairing during meiosis, the recombinase DMC1 as a key player was significantly down-regulated in autotetraploid *B. rapa*, which indicated that homologous recombination pathway for meiotic DSBs repair was probably handicapped in autotetraploid *B. rapa* as numerous chromosome univalents were cytologically observed at the meiotic metaphase I in comparison with diploids. Moreover, the expression of the DMC1 was not dependent on the activity of RAD51 (Da Ines et al., 2013), and RAD51 activity would be initially down-regulated by HED1 during meiosis to enable the activity of DMC1 in yeast (Liu et al., 2014). Alternatively, meiotic DSBs repair mediated by the RAD51-promoted intersister-chromatid recombination was essentially maintained (De Muyt et al., 2009), which could counteract adverse effects raised by down-regulation of DMC1 in autotetraploid *B. rapa*, as it was normally regulated at a transcription level and chromosome fragments were rarely observed cytologically, which frequently occurred in the complete deficiency of meiotic DSBs repair (Kurzbauer et al., 2012; Da Ines et al., 2013).

### Known genes involved in homologs pairing and synapsis in autotetraploid *B. rapa*

The pairing and synapsis of homologous chromosomes during prophase I are necessary for their subsequent orderly segregation at anaphase I during meiosis, and each pair of homologous chromosomes becomes closely associated (synapsed), and establish a cytologically ultrastructure, synaptonemal complex (SC). The gene *ZYP1* encodes a protein for the transverse filaments of the SC (Lynn et al., 2007), and SYN1 as a cohesin protein, is required for sister chromatid arm cohesion and homologous chromosome pairing (Cai et al., 2003). The enhanced expression of *ZYP1* and *SYN1* was observed in the newly formed autotetraploids *A. arenosa* (Yant et al., 2013), and our data showed that transcription levels of *ZYP1* and *SYN1* were also up-regulated in autotetraploid *B. rapa* in contrast with diploids (Table S8), which indicated that the pairing of homologous chromosome was probably enhanced during meiosis due to genomic duplication in autotetraploid *B. rapa*.

## Author contributions

All authors contributed equally to the writing of this manuscript. JB and YY carried out all of the RNA-seq data analysis, conduct chromosomal distribution experiment, and analysis of chromosomal spread results. FW, BT, and XW supervised the project and assisted with analysis of all data. GC and GS supervised the bioinformatics analysis. XZ and HJ assisted with microscopy experiment and ZW assist with RNA preparation for RNA-seq.

### Conflict of interest statement

The authors declare that the research was conducted in the absence of any commercial or financial relationships that could be construed as a potential conflict of interest.
