# Supplementary material for: Transcriptome Analysis of Floral Buds Deciphered an Irregular Course of Meiosis in Polyploid Brassica rapa
Source: Front Plant Sci. 2017 May 12;8:768. doi: 10.3389/fpls.2017.00768 (PMC5427127; doi:10.3389/fpls.2017.00768)
Supplement: Supplementary file 9 [file DataSheet1.DOCX]

**Transcriptome analysis of floral buds deciphered an irregular course of meiosis in polyploid *Brassica rapa***

Janeen Braynen^1, 2#^, YanYang^1#^, FangWei^1*^, Gangqiang Cao^1^, GongyaoShi^1^,Baoming Tian^1*^Xiaowei Zhang^2^, Hao Jia^1, 2^, Xiaochun Wei^2*^, Zhenzhen Wei^1, 2^

* **Corresponding authors:** Fang Wei, E-mail address: [fangwei@zzu.edu.cn](mailto:fangwei@zzu.edu.cn); and Baoming Tian, E-mail address: [tianbm@zzu.edu.cn](mailto:tianbm@zzu.edu.cn); and Xiaochun Wei, E-mail: [jweixiaochun@126.com](mailto:jweixiaochun@126.com).


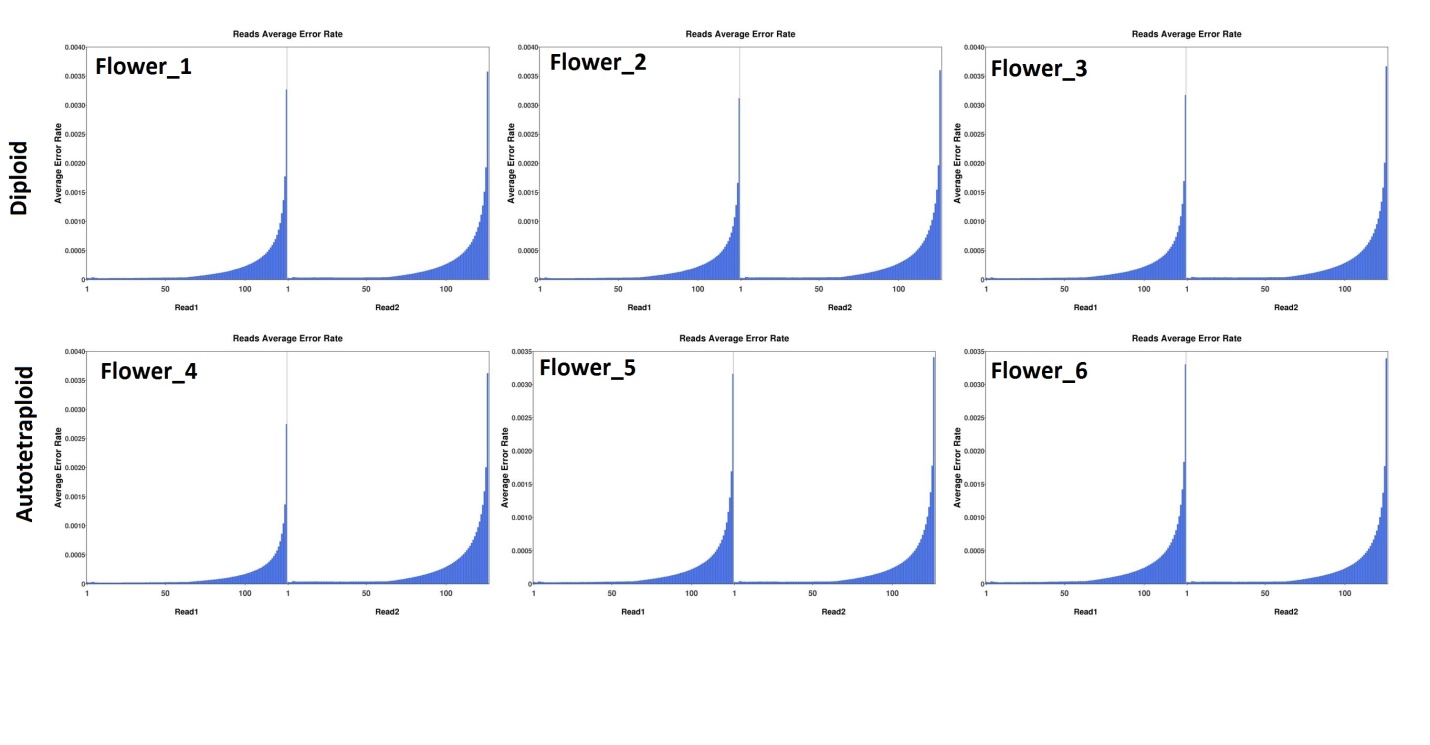


**SUPPLEMENTARY FIGURE 1 |** Quality control for each base in reads 1 and 2 of each sample set displayed by the FASTQC database. Average error rate of read represented by the y-axis and read coverage on the x-axis.


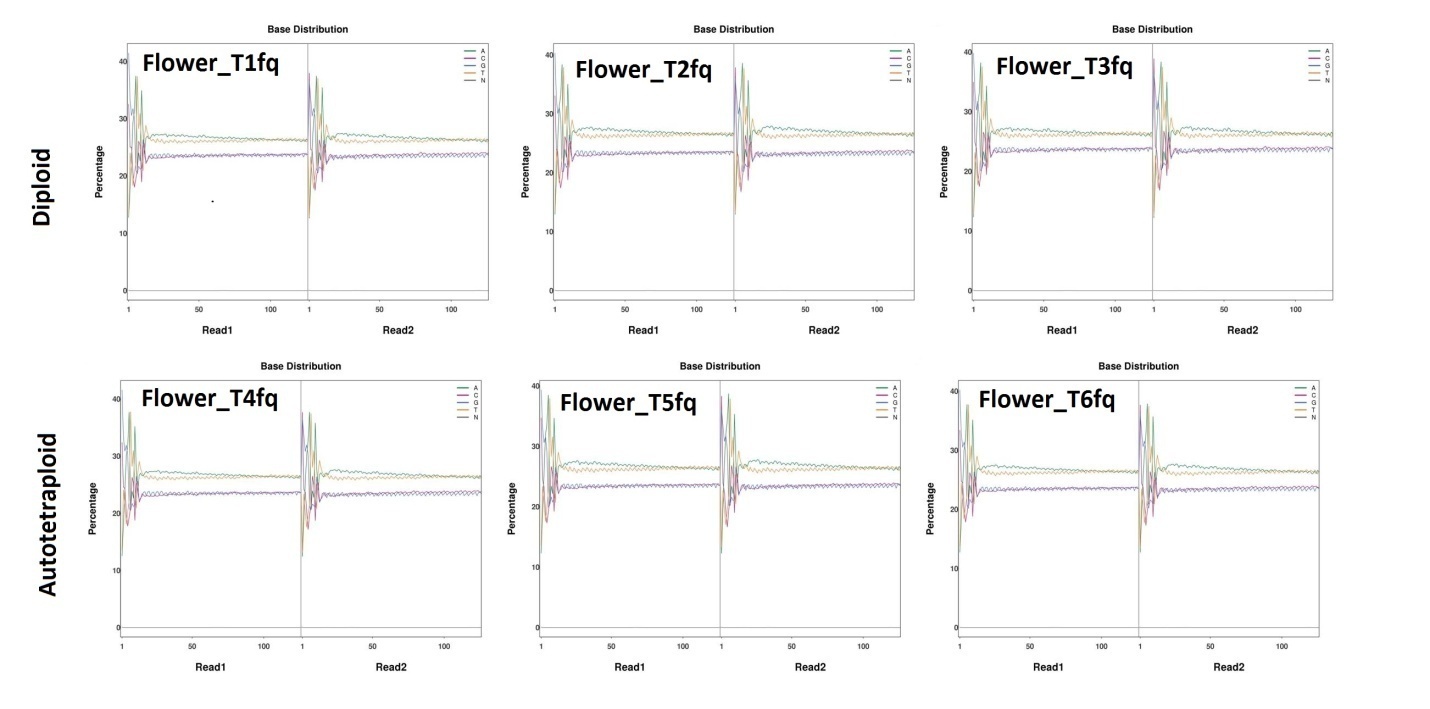


**SUPPLEMENTARY FIGURE 2 |** Base distribution of reads displayed by FASTQC database. The y–axis represents the percentage of bases distributed and the x-axis the number of reads.


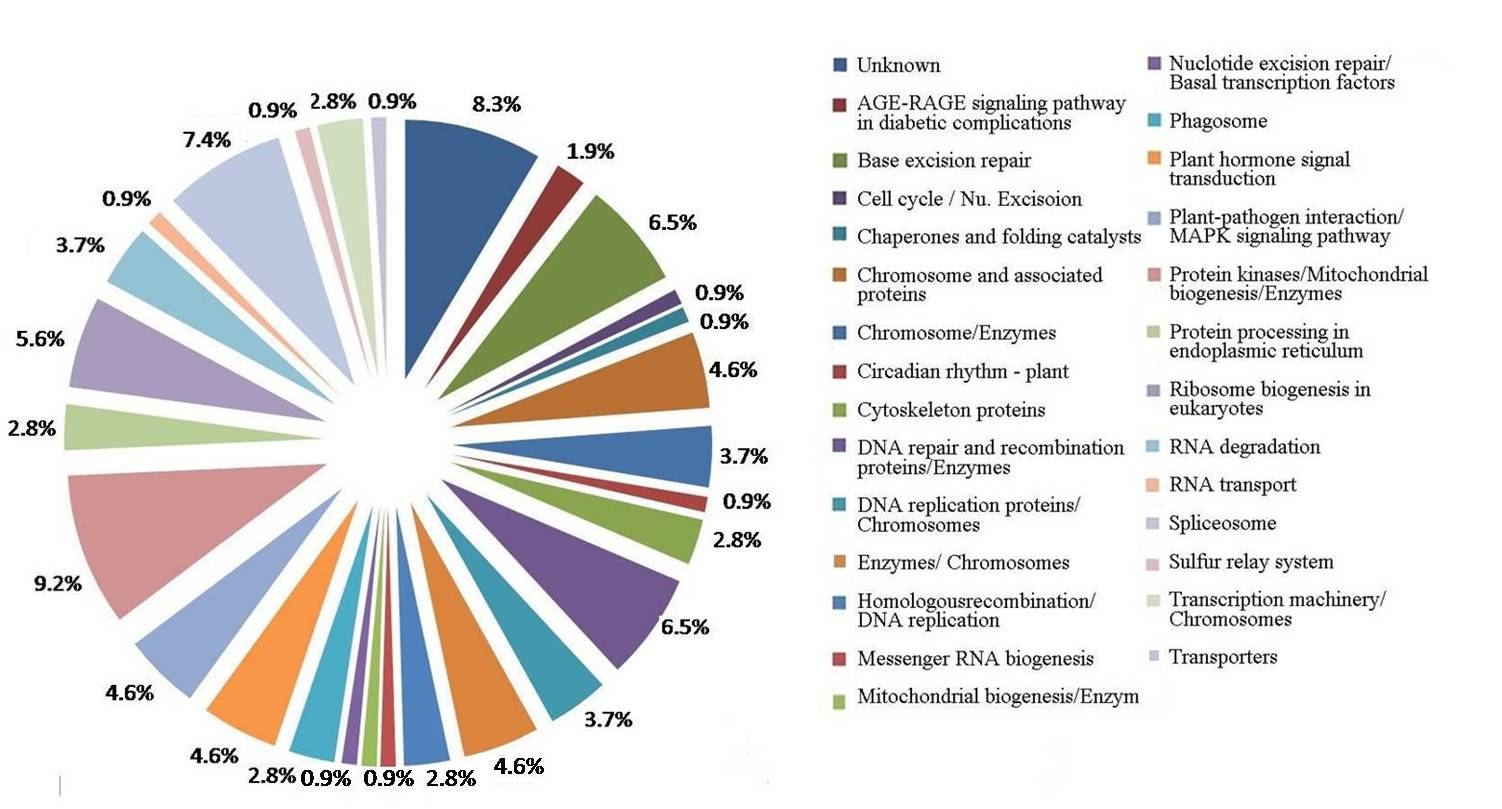


**SUPPLEMENTARY FIGURE 3 |** Classification of meiosis related DEGs. Among 288 DEGs related to meiosis, 108 were assigned to 28 KEGG pathways.


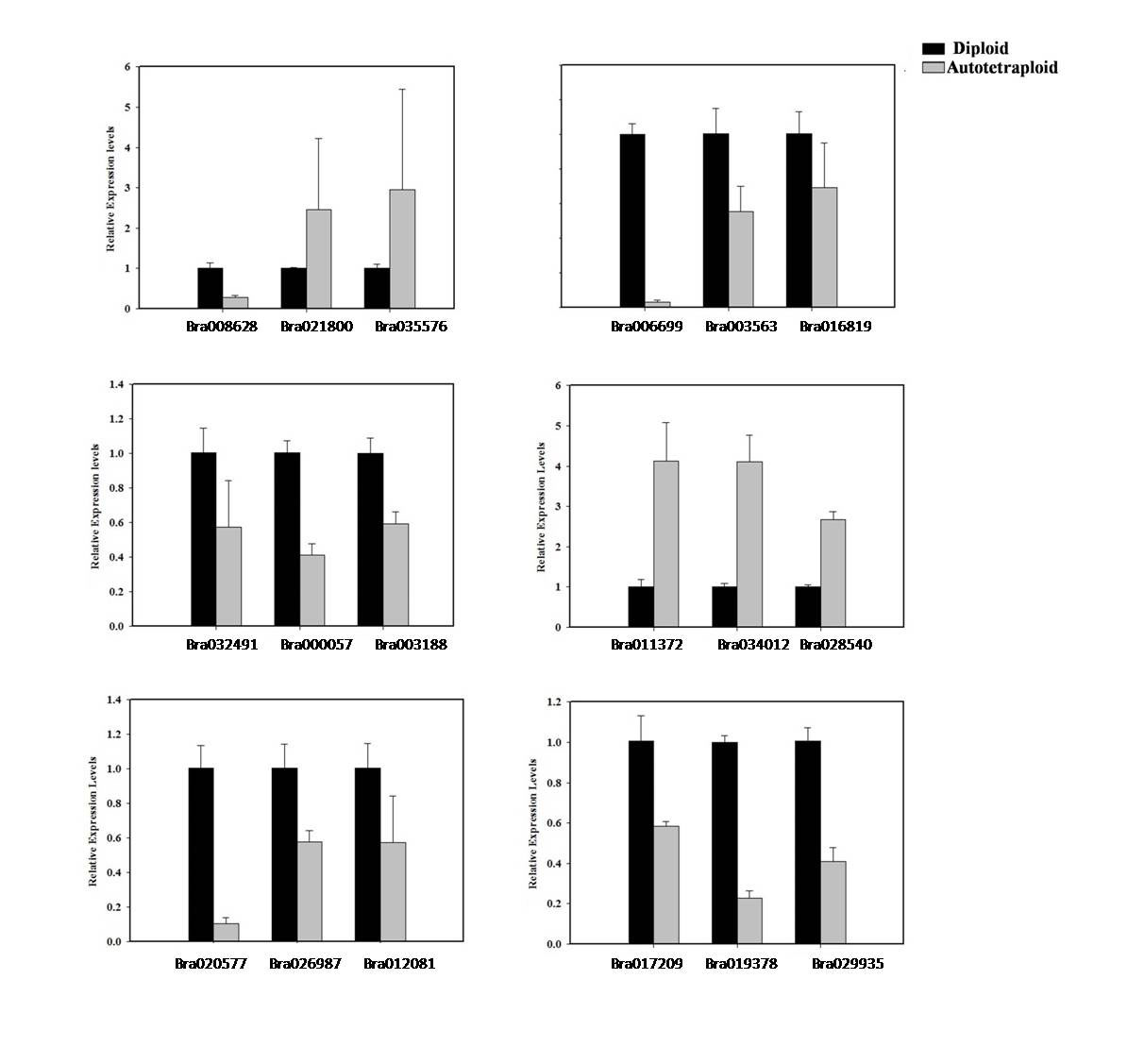


**SUPPLEMENTARY FIGURE 4 |** qRT-PCR confirmation of 18 candidate genes known or related to meiosis between the diploid and autotetraploid.

**
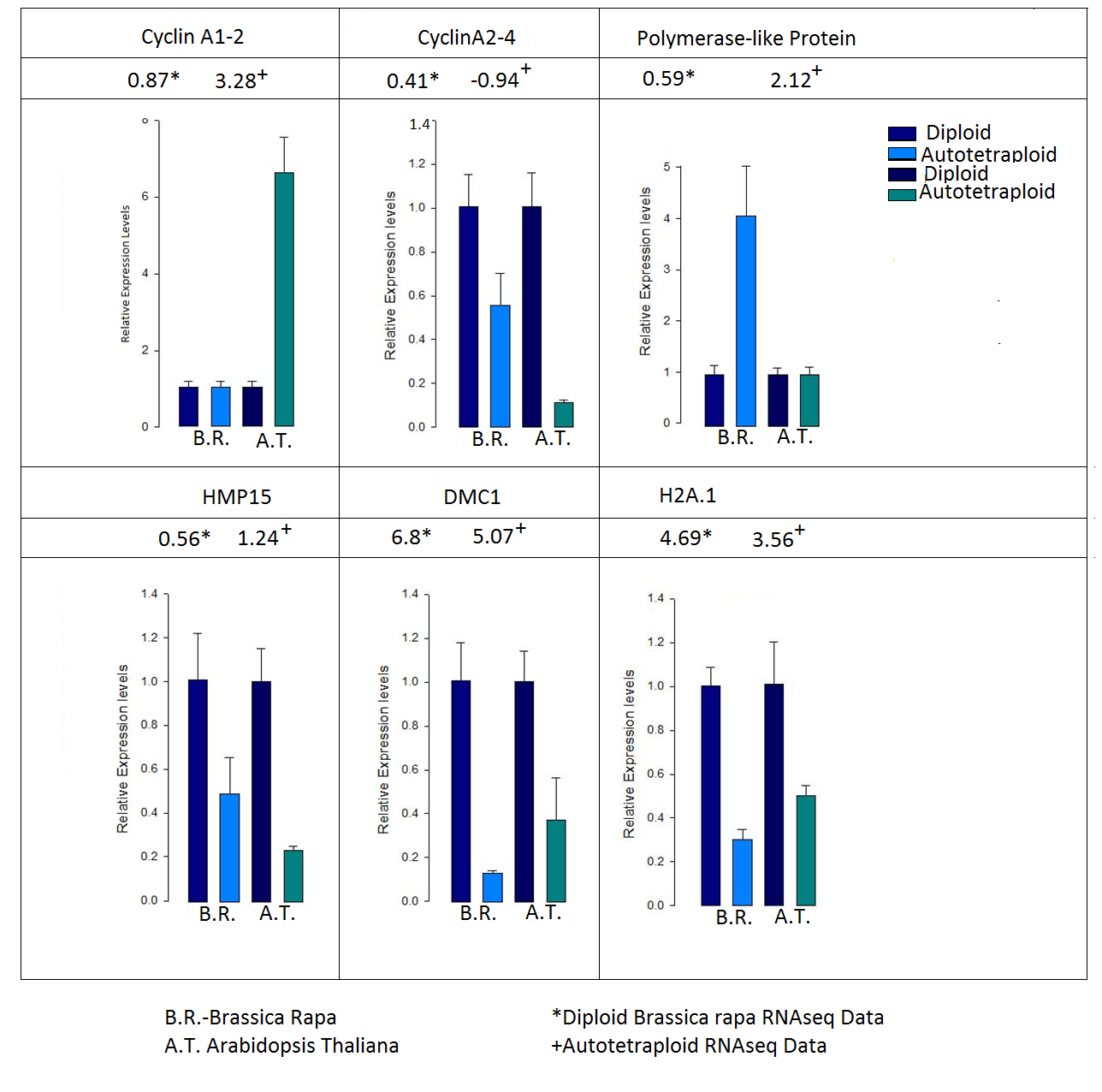
**

**SUPPLEMENTARY FIGURE 5 |** Relative gene expression of *B.rapa* homologs in the *A.thaliana* model species using qRT-PCR.
